# The COVID19 pandemic crisis and the relevance of a farm-system-for-nutrition approach

**DOI:** 10.1007/s12571-020-01071-6

**Published:** 2020-07-09

**Authors:** R. V. Bhavani, R. Gopinath

**Affiliations:** Agriculture Nutrition Health Programme at M S Swaminathan Research Foundation, Chennai, India

**Keywords:** COVID19, Farm-system-for-nutrition, FSN, Malnutrition, Resilient food systems, Sustainable food systems

## Abstract

The Covid19 pandemic should be seen as a wake-up call for humanity, to reflect, rethink and redesign food systems that are safe, healthy, sustainable, and beneficial to all. This crisis has disrupted food supply chains, affecting lives and livelihoods. Hunger and malnutrition is expected to increase and the poor and vulnerable will suffer the most. There is urgent need to build resilient food systems. A location specific farm-system-for-nutrition approach, based on sustainable use of natural resources and local agri-food value chains can help improve household diet diversity and address nutrition deficiencies. The food-based approach can improve preparedness and resilience of communities to withstand the challenge posed by crises in general, and COVID19 in particular.

## Introduction

A large segment of the population in developing countries of the world across Asia, Africa and Latin America depend on agriculture and associated activities for their sustenance. Agriculture covering crops, livestock, aquaculture, fisheries and forests is the world’s biggest employer and the main source of food and income for the extreme poor (FAO 2018). The developing countries of the world are also home to a large population of malnourished people. India is a typical example with more than 50 per cent of its workforce engaged in agriculture, a large majority being small land holders, and prevalence of high levels of child undernutrition and micronutrient deficiency. The last decade has also seen the development of active discourse on leveraging agriculture for nutrition and the need for sustainable food systems in order to achieve the Sustainable Development Goals (SDGs). The COVID19 pandemic that has gripped the world in recent months constitutes a major challenge to food and nutrition security of vulnerable populations. Many countries declared generalised lockdowns as a measure to counter the spread of the disease. Restrictions on transport and movement following the lockdown led to the breakdown of food supply chains, delaying harvest, damaging perishable produce and causing loss to farmers and fishers. Different aspects of the impact on lives and livelihoods of the poor have been documented at global and national levels (FAO 2020; iPES FOOD 2020; MSSRF 2020). It is expected that hunger and malnutrition will increase if necessary measures are not taken urgently (FSIN 2020; UN 2020). According to UN (2020), “The latest data shows that the food security of 135 million people was categorised as crisis level or worse. That number could nearly double before the end of the year due to the impacts of COVID-19”.

## Responding to the COVID19 pandemic from a smallholder perspective

The extent of impact of the COVID19 pandemic therefore makes it all the more important to build resilience within some of the most affected communities, like the smallholder farmers in developing countries, to withstand such shocks and invest in preparedness. The pandemic is the last addition to the list of disasters and crises caused by adverse weather conditions, natural hazards, economic shocks, and conflicts that can upset the livelihood and food and nutrition security of vulnerable populations. FAO has identified four areas or pillars in the framework developed for disaster risk reduction to reduce the impact of disasters on food and nutrition security – enabling environment, early warning and safeguards, prevention and mitigation measures, and preparedness to respond (FAO 2013). The framework is relevant for addressing the COVID19 crisis, which has exposed the ‘fragility of the world’s food and land use system’ (Davey and Steer 2020). Resilient food systems that are based on the sustainable use of natural resources must be promoted as a prevention and mitigation measure against such crises. Food systems have been defined as ‘the entire range of actors and their interlinked value-adding activities involved in the production, aggregation, processing, distribution, consumption and disposal of food products that originate from agriculture, forestry or fisheries, and parts of the broader economic, societal and natural environments in which they are embedded’ (FAO 2018). These need to be accompanied by an enabling policy environment and response preparedness. This calls for agriculture to break away from the thrust on just production, productivity and short-term profit, and instead to mainstream the nutritional dimension, and to promote the diversity of production, while paying attention to soil health and sustainable land use. Farm-system-for-nutrition (FSN) is an example of such a food-based, nutrition-sensitive approach. The FSN approach is defined by M. S. Swaminathan as: *“The introduction of agricultural remedies to the nutritional maladies prevailing in an area through mainstreaming nutritional criteria in the selection of the components of a farming system involving crops, farm animals and wherever feasible, fish”.* (Nagarajan et al. 2014)

## The farm-system-for-nutrition approach

FSN is a location-specific, inclusive approach, with a design based on available resource endowments and specific environment, to address the nutritional needs of small holder farm families. Awareness of balanced diet, nutrient content in different foods and leveraging agriculture for nutrition is an integral component of the approach. In essence, FSN calls for the promotion of location-specific farm systems that integrate arable farming, horticulture, backyard farming and animal farming, to sustainably improve household availability of nutrition while also mitigating risks and conserving natural resources. In developing the farm system design, feasible agricultural interventions to address nutritional deficiencies of the household / community/ location would have to be incorporated. In the words of M. S. Swaminathan, “.....the design of the farming system [can] include specific crop varieties that can address the identified deficiencies. Sweet potato might provide vitamin A, drumstick tree (*moringa olifera*) and *Amaranthus sp.* could address the lack of iron” (Rao and Swaminathan 2017). In addition, the approach recognises the need for other direct interventions – to improve production and market linkages for nutritious crops and indirect interventions – to improve women’s empowerment, nutrition, education, water, sanitation and hygiene (WASH), and natural resource management, along the pathway from agriculture to nutrition (Shetty 2015).

A FSN study on these lines was conducted in two agro-ecologically different locations in India from 2013 to 2018, covering a population of about 1200 households with 5000 people^1^. The study population was dependent on rainfed agriculture and high level of undernutrition prevailed. Diet survey and survey of food consumption pattern revealed that the diet of people was cereal dominated with consumption of all other food groups being lower than the recommended dietary intake levels (Bhaskar et al. 2017). The FSN design focused on increasing the availability and access to nutrient-rich foods by promoting: the cultivation of millets and pulses suited to the region; nutrition-oriented gardens of fruits and vegetables that grow locally; access to animal foods through poultry and fishery; and nutrition awareness. The viability of recommended practices in terms of economic return was demonstrated through on-farm trials, demonstrating the potential for adoption by farmers and the sustainability of the intervention (Pradhan et al. 2019). An endline survey in late 2017, three years after the intervention, revealed an increase in availability and consumption of millets, pulses and vegetables by households, an increase in proportion of households consuming more than 70 per cent of recommended allowance of all food groups, and an improvement in household dietary diversity (MSSRF 2019). In addition to being nutrient dense, millets and pulses are also regarded as climate-resilient crops that are suited to rainfed farming conditions. Building on the experience, agricultural universities in different states of India are now being engaged with, to set up demonstrations of the FSN approach (Bhavani 2018). More work is required to address post-harvest handling and processing and develop local agri-food value chains.

## Conclusion

The crisis created by the COVID19 pandemic brings into focus the relevance of location specific nutrition sensitive agriculture approaches such as FSN that draw on local food diversity. The crisis has also highlighted the importance of decentralized models and of local value chains. In many parts of India for instance, farmers’ producer organisations were seen to be playing a proactive role in aggregating produce from smallholder farmers and facilitating market access (Rengalakshmi and Rao 2020). Pingali et al. (2019) draw attention to the need for similar measures, in their recent work on transforming food systems. The report on Global Food Crises 2020 dwells on this aspect in the light of the COVID19 crisis, and calls for support to ensure the continuous functioning of local food markets, value chains and agri-food systems in food crisis contexts, including support to food processing, transport, marketing and strengthening of local producers’ groups (FSIN 2020). Torero (2020) calls for sustainable use of land and water resources to grow essential, nutritious food in a more resilient way and ‘better treatment for smallholders and migrant workers, who form the backbone of farming’. Swaminathan (2020) draws parallels with the Irish famine crisis of the 1840s and emphasizes the need for genetic diversity for resilient agriculture systems and post-harvest processing, storage, value addition and marketing mechanisms.

The Covid19 pandemic should be seen as a wake-up call for humanity, to reflect, rethink and redesign food systems that are safe, healthy, sustainable, and beneficial to all. A farm-system-for-nutrition approach that is location specific, that promotes production of safe and healthy food, which is based on the sustainable use of natural resources by small farmers, that takes into account the risks posed by climate change, and that promotes development of local value chains, can help build resilience of local communities to crises such as the COVID19 pandemic.

## Footnotes

1 The study was undertaken by the M S Swaminathan Research Foundation under the research programme on Leveraging Agriculture for Nutrition in South Asia (LANSA) http://59.160.153.187/content/farming-system-nutrition accessed 8 May 2020
